# Fetal Cytomegalovirus Infection in the Absence of Maternal Cytomegalovirus-IgM Seropositivity

**DOI:** 10.1007/s43032-024-01487-x

**Published:** 2024-02-22

**Authors:** Hakan Erenel, Guray Tuna, Verda Alpay, İbrahim Polat

**Affiliations:** Perinatology Department, Başakşehir Çam and Sakura City Hospital, 34480 Istanbul, Turkey

**Keywords:** Cytomegalovirus, Fetus, Primary infection, Secondary infection, Serology, Seropositivity

## Abstract

The aim of this study was to evaluate maternal serological status and fetal sonographic findings of Cytomegalovirus (CMV) infection. This is a retrospective study performed at Perinatology Department of Istanbul Başakşehir Çam and Sakura City Hospital. A computerized search was conducted to identify cases who underwent prenatal diagnosis of fetal CMV infection between September 2020 and December 2023. We identified nine cases with fetal CMV infection. The clinical data of the patients, gestational age at the time of diagnosis, serological, sonographic findings, and pregnancy outcomes were analyzed. A computer search of the database was made for the seroprevalance of CMV-IgM and CMV-IgG in our population. The CMV-IgM and IgG results of the 1235 patients who underwent CMV screening in the first trimester between September 2020 and December 2023 were evaluated. Fetal CMV infection was identified in nine patients. None of the 9 cases showed maternal CMV-IgM positivity. Seven of the 9 patients showed high IgG avidity index. Pregnant population had 98 % positivity for CMV-IgG. The evaluation of serologic tests for CMV is not straightforward in the second and third trimester. IgM and IgG avidity should be interpreted with caution in the second and third trimester. In the presence of ultrasound findings suggesting fetal CMV infection and CMV-IgG positivity, invasive diagnostic tests rather than serological test should be discussed with the patient, and non-primary infections should always be considered to minimize overlooked fetal cytomegalovirus infections and missed antiviral treatment opportunity.

## Introduction

Cytomegalovirus (CMV) is the most common cause of congenital infection, with a frequency between 0.2% and 2.2% of all live births [1, 2]. The clinical manifestations of congenital CMV infection include hearing loss, visual impairment, and developmental disabilities [3]. Transmission of CMV can occur due to a maternal primary or secondary (reactivation or reinfection) infection in women with preconceptional immunity [4]. There is a reflex to perform maternal CMV serology in the presence of ultrasound abnormalities such as fetal growth restriction, hyperechogenic bowel, ventriculomegaly, oligohydramnios, and ascites. In practice, positive CMV serology is defined as positive IgM plus low IgG avidity index [5]. The interpretation of serological results of CMV results may be conflicting in the second and third trimester. Here, we report nine cases with fetal CMV infection in the absence of CMV-IgM positivity even as early as at 19 weeks of gestation.

## Materials and Methods

This is a retrospective study performed at Perinatology Department of Istanbul Başakşehir Çam and Sakura City Hospital. A computerized search was conducted to identify cases who underwent prenatal diagnosis of fetal CMV infection and CMV-IgM and IgG screening during pregnancy between September 2020 and December 2023. The study was approved by the local ethical committee. We identified nine cases with fetal CMV infection. In all cases, gestational age on admission, reason for referral, and gestational age at diagnosis were noted. Sonographic evaluations were performed with ARIETTA 850 (Hitachi Medical Corporation, Tokyo, Japan) device (3.5-mHz abdominal and 5-mHz vaginal transducers). Samples of serum were tested for CMV-specific IgG and IgM antibodies using IgG and IgM Elecsys kit by electrochemiluminescence immunoassay technique (Cobas e801 analyzer, Roche Diagnostic GmbH, Mannheim, Germany). Results obtained with the Cobas CMV-IgM assay was interpreted as follows: non-reactive: < 0.7 cutoff-index (COI), indeterminate: ≥ 0.7 to < 1.0 COI, reactive: ≥ 1.0 COI. Cobas CMV-IgG assay was interpreted as follows: non-reactive: < 0.5 U/mL, indeterminate: ≥ 0.5 to < 1.0 U/mL, reactive: ≥ 1.0 U/mL. CMV-IgG avidity test was performed in patients who had positive IgG results. The IgG avidity was measured by the Vidas method (bioMérieux, France). An avidity index <0.40 was considered low and >0.65 high. We recommend amniocentesis and CMV PCR analysis in all cases in the suspicion of infection according to ultrasound findings regardless of serologic results. Amniocentesis and CMV PCR analysis performed after 20 weeks of gestation in all patients. Quantitative CMV PCR assays were performed using DNA extraction kit (Qiagen, Hilden, Germany). The assay was carried out in a Rotor-Gene Q 5PLEX instrument (Qiagen, Hilden, Germany)

All patients underwent detailed ultrasound examination. All ultrasound examinations were performed by one experienced maternal and fetal specialist (H.E). Fetal growth restriction (FGR) was diagnosed when the abdominal circumference was below the 5th percentile. Placentomegaly was defined by a placental thickness of more than 4 cm. The intestines which have echogenicity equal to bone were defined as hyperechogenic bowel. Magnetic resonance imaging was performed in the presence of intracranial findings. Pediatric neurology consultation was made in regard to prognosis of CMV infection. The choice of termination of pregnancy was discussed with the patients after the positive amniotic fluid CMV PCR test. Fetopsy was performed after termination of pregnancy according to decision of parents. The clinical data of the pregnant women, gestational age at the time of diagnosis, serological, sonographic findings, and pregnancy outcomes were analyzed.

## Results

Between September 2020 and December 2023, fetal CMV infection was identified in nine patients. The clinical characteristics, ultrasound (US) findings, and maternal serology are shown on Table 1. Ventriculomegaly was the most common indication for referral. Synechia in the occipital horn was the most common ultrasound finding. None of the nine cases showed maternal CMV-IgM positivity. Seven of the 9 patients showed high IgG avidity index. Avidity index was unavailable in two patients. Seven of the 9 patients opted for termination of pregnancy. A computerized search for CMV screening showed CMV-IgG positivity in 1210 (98%) of the 1235 patients and CMV-IgM positivity 60 of the 1235 (0.5%) patients.

**Table 1:** Characteristics of 9 fetuses with US findings and maternal serology

Case	GA at first examination (week+day)	Indication for referral	US findings	GA at serology testing	CMV serology			Amniocentesis CMV DNA PCR* (IU/mL)	Outcome
					IgM(COI)	IgG(U/mL)	Avidity		
1	19+5	Ascites	HC <3p, FL <3p, ascites, placentomegaly, hyperechogenic bowel, FGR, increased MCA PSV (1.98 MoM)	19+5	Negative (0.331)	Positive (>500)	High (0.82)	1227858	TOP
2	30+3	Short femur	FGR	30+3	Negative (0.172)	Positive (>500)	n.a.	n.a.	Live Birth
	36+2	FGR	Severe FGR, periventricular calcifications, mild pericardial effusion	36+2	Negative (0.161)	Positive (>500)	High (0.84)		
3	27+0	FGR, microcephaly	AC 13p, HC between −2SD and −1 SD Synechia in the occipital horn, increased cardiothoracic ratio	27+0	Negative (0.170)	Positive (>500)	n.a.	n.a.	Live Birth
4	23+6	Ventriculomegaly	Bilateral synechia in the posterior horn, periventricular hyperechogenicity	23+6	Negative (0.155)	Positive (>500)	n.a.	8354099	TOP
5	24+0	Mild ventriculomegaly	Mild ventriculomegaly (11 mm), HC -2SD, ventricular synechia in the posterior horn, periventricular calcifications	24+0	Negative (0.306)	Positive (>500)	High (0.65)	2434538	TOP
6	20+1	Echogenic bowels, oligohydramnios, ascites	Echogenic bowels, oligohydramnios, ascites, placentomegaly, megacisterna magna, increased MCA PSV (1.62 MoM), synechia in the occipital horn and echogenic periventricular halo in the brain	20+1	Negative (0.161)	Positive (>500)	High (0.94)	35284003	TOP
7	30+2	Low HC	Microcephaly, megacisterna magna, abnormal cortilcal development suggestive of lissencephaly, periventricular calcifications, bilateral synechia in the occipital horn and increased MCA PSV (1.97 MoM), hepatomegaly	30+2	Negative (0.362)	Positive (>500)	High (0.91)	16714913	TOP
8	21+4	Ventriculomegaly, megacisterna magna	Severe bilateral ventriculomegaly ( 16 mm), periventricular halo, cerebellar hypoplasia, dysgenetic corpus callosum, delayed sylvian fissure operculization, dilated third ventricle, grade 4 intraventricular hemorrhage and periventricular cysts	22+2	Negative (0.655)	Positive (312)	High (0.91)	19495749	TOP
9	25+4	Megacisterna magna	Bilateral mild ventriculomegaly, bilateral synechia in the occipital horn, periventricular halo, hepatosplenomegaly, increased MCA PSV (2.66 MoM)	25+4	Negative (0.384)	Positive (414)	High (0.85)	21237352	TOP

*AC* abdominal circumference, *CMV* cytomegalovirus, *HC* head circumference, *FL* femur length, *US* ultrasound, *FGR* fetal growth restriction, *MCA* middle cerebral artery, *PSV* peak systolic velocity, *SD* standard deviation, *TOP* termination of pregnancy. *Minor detection limit 69.7 IU/ml

### Case 1

**Fig. 1:**
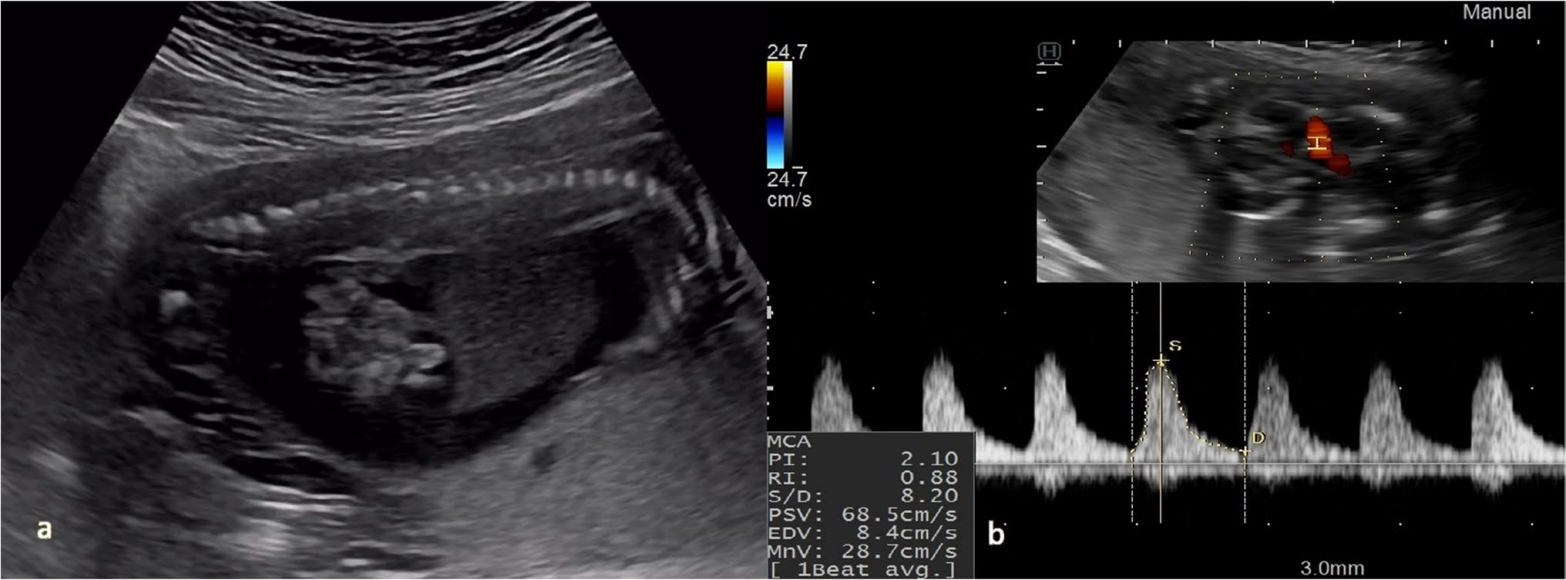
**a** Fetal ascites, hyperechogenic bowel. **b** Elevated middle cerebral artery peak systolic velocity indicates fetal anemia

Case 1 was referred at 20 weeks of gestation due to fetal ascites. Detailed ultrasound examination showed ascites, hyperechogenic bowel, increased middle cerebral artery peak systolic velocity (1.98 MoM), (shown in Fig. 1a, b), fetal growth restriction, and placentomegaly. Maternal serology was negative for toxoplasmosis and parvovirus B19. Maternal serology was negative for CMV-IgM and positive for CMV-IgG with high IgG avidity index. Amniocentesis was performed, and CMV PCR was positive in the amniotic fluid. According to the request of the patient, the pregnancy was terminated. Fetopsy was performed. The postabortal gross examination showed petechial lesions on the skin. Immunohistochemical staining was positive for CMV in the liver, kidneys, and placenta.

### Case 2

**Fig. 2:**
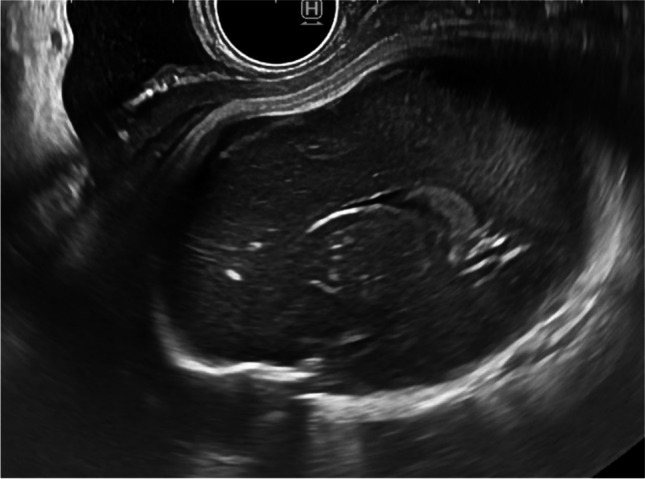
Parenchymal calcifications in a parasagittal plane of the brain

Case 2 was referred at 31 weeks of gestation due to short femur to our perinatology unit. A diagnosis of FGR was made and maternal serology was negative for CMV-IgM and positive for CMV-IgG. IgG avidity index was unavailable. At 36 weeks of gestation, a second examination showed periventricular calcifications (shown in Fig. 2), severe fetal growth restriction (EFW:1875 gr), and low head circumference (1 percentile). A repeat maternal serology testing showed high IgG avidity index. The patient declined invasive diagnostic tests. At 37 weeks of gestation, a female infant was born with a birth weight of 2030 g, a length of 51 cm, a head circumference of 33 cm, and Apgar scores of 8/9/10. Physical examination and blood count parameters of the newborn were usual. Hearing and ophthalmological tests were normal. Result of urine PCR for CMV was positive and cranial US confirmed our findings. The infant was treated with valganciclovir for 12 months.

### Case 3

**Fig. 3:**
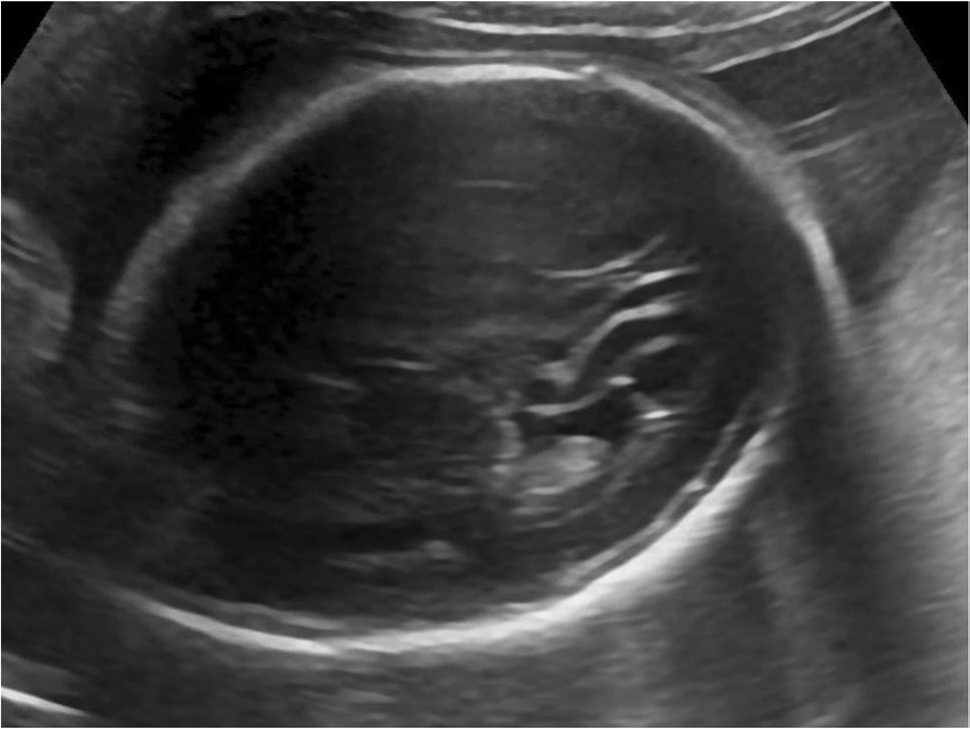
Synechia in the posterior horn

Case 3 was referred at 27 weeks of gestation. Due to suspicion of microcephaly and FGR, detailed ultrasound examination showed low head circumference (between −2SD and −1 standard deviation) and ventricular synechia in the posterior horn (shown in Fig. 3). Maternal serology was negative for CMV-IgM and positive for CMV-IgG. IgG avidity index was unavailable. Amniocentesis was performed, and CMV PCR was positive in the amniotic fluid. Termination of pregnancy was discussed, and the patient chose to continue the pregnancy. At 38 weeks of gestation, a female infant was born with a birth weight of 2780 g, a length of 50 cm, a head circumference of 33 cm, and Apgar scores of 7/9/10. Physical examination and blood count parameters were usual. Hearing and ophthalmological tests were normal. Result of urine PCR for CMV was positive. The infant was treated with valganciclovir for 12 months.

### Case 4

**Fig. 4:**
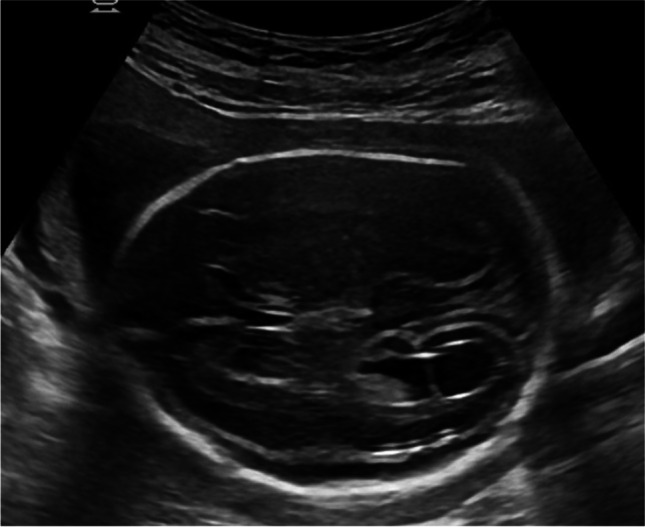
Synechia in the posterior horn and periventricular echogenic halo

Case 4 was referred at 24 weeks of gestation due to mild ventriculomegaly. Detailed ultrasound examination showed bilateral ventricular synechia in the posterior horn and periventricular echogenic halo (shown in Fig. 4). Maternal serology was negative for CMV-IgM and positive for CMV-IgG. IgG avidity index was unavailable. Amniocentesis was performed and CMV PCR was positive in the amniotic fluid. According to the request of the patient, the pregnancy was terminated. The patient declined fetal autopsy.

### Case 5

**Fig. 5:**
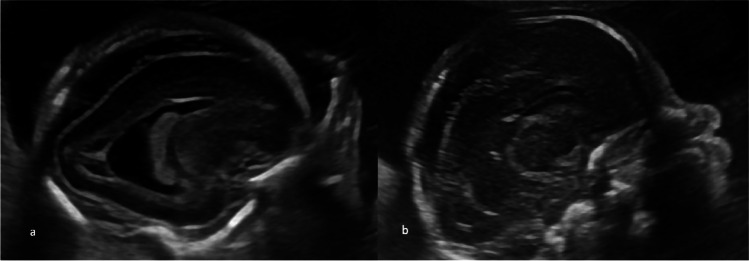
**a** Synechia in the posterior horn and periventricular echogenic halo in parasagittal plane. **b** Parenchymal calcifications in a parasagittal plane of the brain

Case 5 was referred at 24 weeks of gestation due to mild ventriculomegaly. Fetal neurosonography confirmed this finding and additionally revealed unilateral ventricular synechia in the posterior horn and periventricular calcifications (shown in Fig. 5a, b). Head circumference was on −2 standard deviation. Maternal serology was negative for CMV-IgM and positive for CMV-IgG with high avidity index. Amniocentesis was performed, and CMV PCR was positive in the amniotic fluid. According to the request of the patient, the pregnancy was terminated. The patient declined fetal autopsy.

### Case 6

**Fig. 6:**
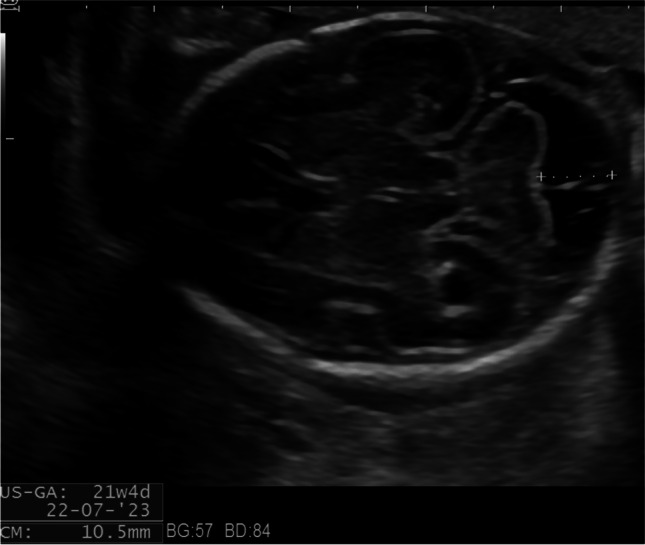
Megacisterna magna

Case 6 was referred at 20 weeks of gestation because of fetal echogenic bowels, oligohydramnios, and ascites. Detailed fetal examination confirmed these findings and additionally revealed increased middle cerebral artery peak systolic velocity (1.62 MoM), megacisterna magna (shown in Fig. 6), synechia in the posterior horn, and echogenic periventricular halo in the brain. Maternal serology was negative for CMV-IgM and positive for CMV-IgG with high IgG avidity index. Amniocentesis was performed, and CMV PCR was positive in the amniotic fluid. According to the request of the patient, the pregnancy was terminated. The patient declined fetal autopsy.

### Case 7

**Fig. 7:**
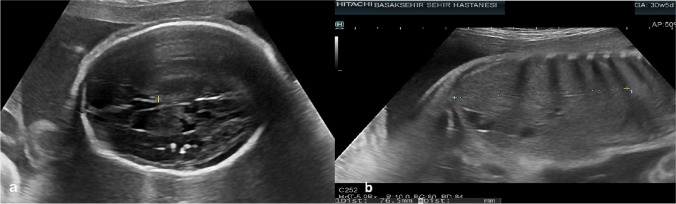
**a** Abnormal cortical development suggestive of lissencephaly and parenchymal calcifications. **b** Hepatomegaly

Case 7 was referred at 30 weeks of gestation because of low head circumference. Detailed cranial examination revealed microcephaly, megacisterna magna, periventricular calcifications, bilateral synechia in the posterior horn, increased middle cerebral artery peak systolic velocity (1.97 MoM), and abnormal cortical development suggestive of lissencephaly and hepatomegaly (shown in Fig. 7a, b). Maternal serology was negative for CMV-IgM and positive for CMV-IgG with high IgG avidity index. According to the request of the patient, the pregnancy was terminated. Amniocentesis was performed during fetocide procedure, and CMV PCR was positive in the amniotic fluid. The patient declined fetal autopsy.

### Case 8

**Fig. 8:**
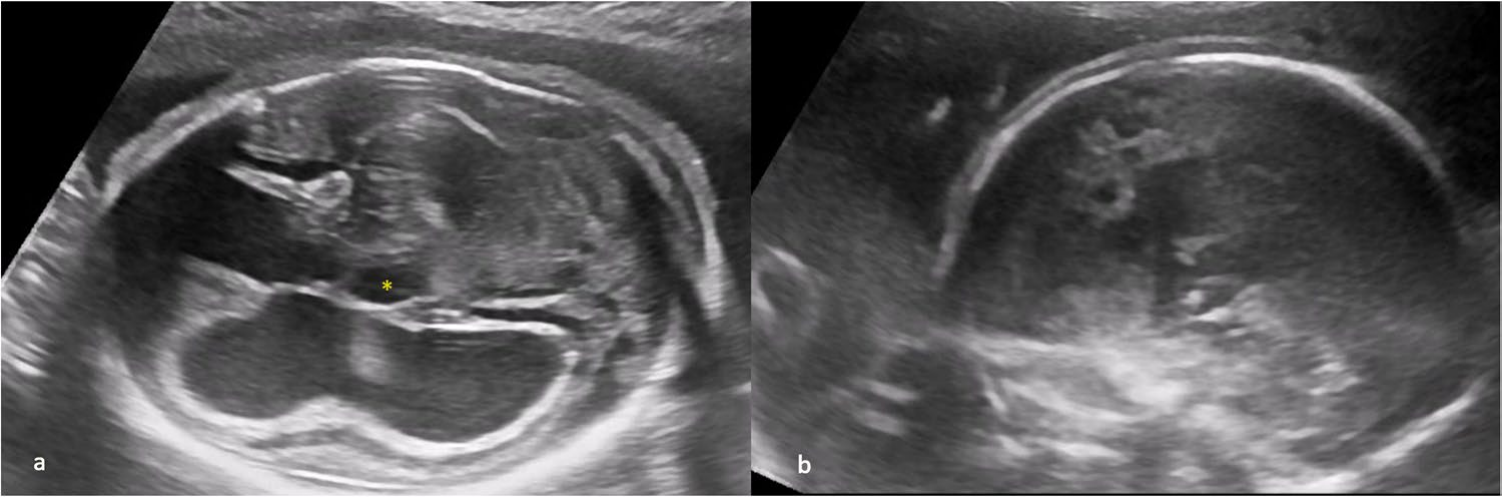
**a** Periventricular halo, delayed Sylvian fissure operculization and dilated third ventricle (*). **b** Periventricular cysts associated with intraventricular hemorrhage (25 weeks of gestation)

Case 8 was referred at 21 weeks of gestation because of ventriculomegaly and megacisterna magna. Detailed examination revealed bilateral severe ventriculomegaly, periventricular halo, cerebellar hypoplasia, and dysgenetic corpus callosum. Maternal serology was negative for CMV-IgM and positive for CMV-IgG with high IgG avidity index. Amniocentesis was performed, and CMV PCR was positive in the amniotic fluid. At 25 weeks of gestation, repeat ultrasound examination showed additional findings such as delayed Sylvian fissure operculization, dilated third ventricle, grade 4 intraventricular hemorrhage, and periventricular cysts (shown in Fig. 8a, b). According to the request of the patient, the pregnancy was terminated. The patient declined fetal autopsy.

### Case 9

Case 9 was referred at 25 weeks of gestation because of megacisterna magna. We observed bilateral mild ventriculomegaly and synechia in the occipital horn, periventricular halo, hepatosplenomegaly, and increased MCA PSV (2.66 MoM). Maternal serology was negative for CMV IgM and positive for CMV IgG with high IgG avidity index. Amniocentesis was performed, and CMV PCR was positive in the amniotic fluid. According to the request of the patient, the pregnancy was terminated. The patient declined fetal autopsy.

## Discussion/Conclusion

In our paper, we would like to present maternal serological findings of the cases with the diagnosis of fetal CMV infection. None of our patients showed IgM positivity which leads us to abandon the perception of ruling out the possibility of vertical transmission in the absence of CMV-IgM positivity [6]. Gunkel et al. published 5 cases with fetal CMV infection, in which congenital CMV infection was ruled out due to negative maternal CMV-IgM [6]. It is difficult to distinguish maternal infection between primary infection or non-primary infections without information regarding to preconceptional immunity. Picone et al. showed very different serological and molecular pattern in non-primary infection such as positive or negative CMV-IgM, significant raise or stability of CMV-IgG, and fluctuant CMV-IgG avidity [7]. In their study, 7 of the 9 cases had CMV-IgM negative. In addition, a rapid drop of IgM levels and appearance of high IgG avidity index even within 90 days after primary infection have been reported which makes interpretation more difficult [8]. Revello et al. showed that maternal IgM antibodies were negative in the 13.6% of the patients after 31–60 days of primary CMV infection. Different commercial kits showed variation in IgM results after 31–60 days and 61–90 days of primary CMV infection [9]. Switching from IgM positivity to negativity might be earlier with regard to commercial kits. Growing evidence shows that a considerable amount of congenital CMV cases are secondary to non-primary infection [10, 11]. Gonce et al. reported 18 pregnancies with confirmed fetal CMV infection, and 10 of the 18 patients (56%) showed negative maternal IgM antibodies at time of the sonographic findings and diagnosis of fetal infection [12]. They did not perform IgG avidity testing. In our study, 7 of the 9 cases showed high IgG avidity index. It is difficult to exclude primary or non-primary maternal CMV infection in the pregnancy when the serological tests have been performed in the second or third trimester and in the absence of preconceptional serological results. Therefore, it should be emphasized that in the presence of ultrasound findings suggesting fetal CMV infection, amniocentesis and real-time analysis of polymerase chain reaction for CMV should be discussed with the patient with CMV IgG positivity regardless of the CMV-IgM positivity and CMV-IgG avidity. In our country, seropositivity for CMV-IgG is more than 95% [13, 14]. Consistent with available literature, we found %98 seropositivity for CMV-IgG. In our study, we can argue that most of fetal CMV infections are secondary to maternal non-primary CMV infections. A larger number of congenital CMV infections were caused by maternal non-primary infection than by primary infection [15]. Diagnosis of non-primary maternal CMV infection may become a more important issue after the growing evidence of reduced cytomegalovirus-related morbidity for newborn after maternal valacyclovir treatment [16, 17].

Major limitation of the study is small sample size. Other limitations of our study are retrospective design and the absence of maternal serologic tests in the preconceptional period and first trimester which provides better discrimination between primary and non-primary CMV infections.

In conclusion, the evaluation of serologic tests for CMV is not straightforward in the second and third trimester. IgM and IgG avidity should be interpreted with caution in the second and third trimester. In the presence of ultrasound findings suggesting fetal CMV infection and CMV IgG positivity, invasive diagnostic tests rather than serological test should be discussed with the patient, and non-primary infections should always be considered to minimize overlooked fetal cytomegalovirus infections and missed antiviral treatment opportunity.

## Data Availability

Not applicable.

## Data Availability

The data supporting this study’s findings are available on request from the corresponding author. The data are not publicly available due to patient privacy and ethical restrictions.
